# Neuroprotective Properties of *Litchi chinensis* and Its Phytochemicals in Preclinical Models of Alzheimer's Disease

**DOI:** 10.1002/brb3.71474

**Published:** 2026-05-14

**Authors:** Emon Mia, Md. Arif Hossain, Asmaul Husna Bristy, Rakib Hossan, Uzma Asif, Asif Khan Sherwani, Mohammad Y. Alshahrani, Samy Selim, Imam Hossen Rakib, Noshin Tasnim Yana, Khadija Akter, Md. Sakib Al Hasan

**Affiliations:** ^1^ Department of Pharmacy Gopalganj Science and Technology University Gopalganj Bangladesh; ^2^ Department of Biochemistry, Medicine Program Batterjee Medical College Jeddah Saudi Arabia; ^3^ Research and Development Unit Jamjoom Pharmaceutical Jeddah Kingdom of Saudi Arabia; ^4^ Central Labs King Khalid University Abha Saudi Arabia; ^5^ Department of Clinical Laboratory Sciences, College of Applied Medical Sciences King Khalid University Abha Saudi Arabia; ^6^ Department of Clinical Laboratory Sciences, College of Applied Medical Sciences Jouf University Sakaka Saudi Arabia; ^7^ Alor Dishari Research Organization Dhaka Bangladesh

**Keywords:** Alzheimer's disease, amyloid‐β, *Litchi chinensis*, neuroprotection, tau hyperphosphorylation

## Abstract

**Purpose:**

Alzheimer's disease (AD) is a progressive neurodegenerative disorder involving amyloid‐β deposition, tau hyperphosphorylation, oxidative stress, and neuroinflammation. This review systematically evaluates the neuroprotective effects of *Litchi chinensis* and its phytochemicals against AD, focusing on modulation of Aβ accumulation, tau pathology, oxidative stress, neuroinflammation, apoptosis, and synaptic dysfunction using available preclinical evidence.

**Method:**

A systematic literature search was conducted in PubMed, Scopus, Web of Science, ScienceDirect, and Google Scholar up to August 2025 using relevant keywords. Studies investigating neuroprotective effects of *Litchi chinensis* extracts or compounds in in vitro or in vivo AD models were included, while unrelated studies, duplicates, abstracts, and non‐full‐text articles were excluded.

**Results:**

Litchi chinensis extracts and phytochemicals demonstrated broad neuroprotective actions. In triple‐transgenic mice, oligonol treatment (0.25–0.50 mg/mL) significantly reduced amyloid precursor protein (APP), β‐secretase, and amyloid‐β levels, while also decreasing tau hyperphosphorylation. Seed extracts (0.7–2.8 g/kg/day) reduced amyloid‐β accumulation and neuronal injury in Sprague–Dawley rats. Anti‐inflammatory effects were evident through decreased tumor necrosis factor‐α, IL‐1β, IL‐6, and interferon gamma, alongside increased IL‐4. Antioxidant defenses were enhanced via upregulation of antioxidant enzymes such as glutathione peroxidase‐1 and superoxide dismutase‐2, while apoptosis was suppressed by increasing Bcl‐2 and reducing Bax and caspase activity. Synaptic integrity was preserved through upregulation of PSD95, synaptophysin, and serotonin receptor proteins, resulting in improved learning and memory in AD models. Additional benefits included enhanced mitochondrial and proteasomal activity, alleviation of endoplasmic reticulum (ER) stress, and induction of neurotrophic factors like insulin‐like growth factor 2 and fibroblast growth factor 21.

**Conclusion:**

*Litchi chinensis* demonstrates a multitargeted neuroprotective role, making it a promising natural therapeutic candidate for Alzheimer's management. However, as most findings are limited to preclinical models, further clinical studies are necessary to validate efficacy, ensure safety, and explore its translational potential.

## Graphical abstract

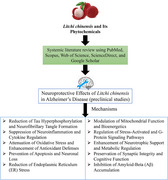

## 1 Introduction

AD is the most common cause of dementia, accounting for 60%–80% of all dementia cases worldwide (Rayathala and Kumar 2022). It is a progressive neurodegenerative disorder characterized by memory impairment, cognitive decline, behavioral disturbances, and ultimately, loss of independence (Sohrabi and Weinborn 2019). AD often overlaps with other neurological disorders, in both symptoms and underlying mechanisms. For example, it shares vascular components with vascular dementia and movement abnormalities with Parkinson's disease (Kalaria 2002). These overlaps complicate diagnosis and highlight the interconnected nature of neurodegenerative conditions, suggesting that common pathways such as oxidative stress, neuroinflammation, and impaired protein clearance contribute to disease progression across multiple disorders (Reynolds et al. 2007; Teleanu et al. 2022).

The global burden of AD has escalated dramatically in recent decades due to aging populations and increased life expectancy. According to the World Health Organization (WHO), in 2019, over 55 million people worldwide were living with dementia. This number is expected to rise to 78 million by 2030 and 139 million by 2050 due to aging populations (World Health Organization 2021). AD contributes to the majority of these cases, making it a major public health concern. Each year, nearly 10 million new cases of dementia are reported globally, with AD being the predominant subtype (World Health Organization 2021).

Around 60% of people with dementia live in low‐ and middle‐income countries, and this share is rising. Dementia is the seventh leading cause of death and a major contributor to disability and dependency in older adults. The global cost was US$1.3 trillion in 2019, projected to reach US$2.8 trillion by 2030 (World Health Organization 2021). Beyond the economic strain, AD imposes a heavy emotional and psychological toll on patients and caregivers, often resulting in caregiver stress, depression, and reduced quality of life (Lavretsky 2005). Developing countries, particularly in Asia and Africa, face unique challenges due to limited healthcare infrastructure, cultural stigma, and insufficient resources for dementia care (Guerchet et al. 2017). As the global population continues to age, the Alzheimer's crisis is projected to intensify, highlighting the urgent need for effective preventive and therapeutic strategies.

At present, therapeutic options for AD remain limited. Approved pharmacological treatments, such as acetylcholinesterase inhibitors (donepezil, rivastigmine, and galantamine) and the N‐methyl‐D‐aspartate (NMDA) receptor antagonist memantine, offer only symptomatic relief without altering disease progression (Hansen et al. 2008; Olivares et al. 2012; Tan et al. 2014). More recently, monoclonal antibodies targeting Aβ, such as aducanumab and lecanemab, have gained conditional approval (Chowdhury and Chowdhury 2023). However, these therapies remain controversial due to limited efficacy, high cost, and safety concerns. Memantine commonly causes central nervous system effects like dizziness, headache, confusion, drowsiness, and insomnia, as well as gastrointestinal issues such as constipation, nausea, vomiting, and loss of appetite. These side effects are generally mild but require monitoring during treatment (Varadharajan et al. 2023). This therapeutic gap underscores the urgent need to explore alternative strategies, including dietary and plant‐based interventions that may act on multiple pathological pathways simultaneously.

Medicinal plants and their phytochemicals have received significant attention in recent years for their potential in preventing and managing neurodegenerative diseases (Kumar and Khanum 2012). Natural products are rich in bioactive compounds such as flavonoids, polyphenols, alkaloids, and terpenoids (Yana et al. 2025), which exhibit antioxidant, anti‐inflammatory, anti‐apoptotic, and neuroprotective properties (Chen et al. 2022). Unlike conventional drugs that often target a single molecular pathway, natural compounds act on multiple signaling cascades, thereby offering a multitargeted approach against the complex pathology of AD (Iqubal et al. 2021). Moreover, plant‐derived compounds are generally considered safe and cost‐effective, making them attractive candidates for long‐term use in the prevention of cognitive decline (Alum et al. 2025).

*Litchi chinensis*, commonly known as lychee, is a subtropical fruit native to Southeast Asia and widely cultivated in China, India, Thailand, and other tropical regions (Soni and Agrawal 2017). Traditionally, lychee seeds and fruits have been used in Chinese medicine for treating various ailments, including digestive disorders, pain, and neurological conditions (Kanwal et al. 2025). Modern phytochemical studies reveal that lychee seeds are rich in bioactive compounds such as procyanidins, catechins, flavanols, and saponins (Anjum et al. 2017). One of the most studied derivatives, oligonol, is a low‐molecular‐weight polyphenol mixture with enhanced bioavailability, exhibiting strong antioxidant and anti‐inflammatory activities (Ahn et al. 2015).

In addition to its pharmacological potential, the safety profile of *Litchi chinensis* is an important consideration for its therapeutic application. Lychee fruit has long been consumed as a dietary component in many Asian countries, suggesting a relatively favorable safety profile when used in moderate amounts (Anjum et al. 2017). Preclinical studies investigating lychee‐derived compounds, such as oligonol and seed polyphenols, have generally reported low toxicity and good tolerability in experimental models at pharmacologically relevant doses (Thirunavukkarasu et al. 2012). Moreover, polyphenolic compounds present in *Litchi chinensis* are widely recognized for their safety and antioxidant benefits in various nutritional and therapeutic contexts (Thirunavukkarasu et al. 2012).

**FIGURE 1:**
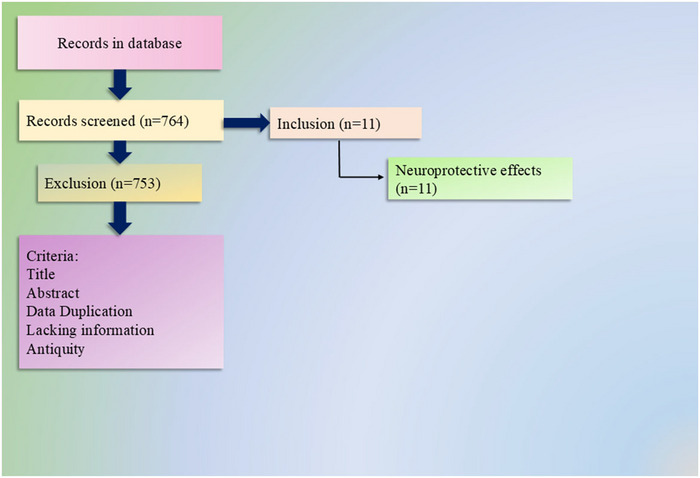
The study selection process used in this research. The illustration was created using a template adapted and modified from a previous article (Al Hasan et al. 2025).

Preclinical evidence suggests that lychee seed extracts and their constituents can exert neuroprotective effects by modulating key pathological processes implicated in AD. These include inhibition of tau hyperphosphorylation via the GSK‐3β signaling pathway, reduction of Aβ accumulation, enhancement of synaptic plasticity, suppression of neuronal apoptosis, and attenuation of oxidative stress and neuroinflammation (Choi et al. 2014; Wang et al. 2017). Both in vitro and in vivo studies have demonstrated that lychee‐derived compounds improve learning, memory, and recognition ability in AD models (Sakurai et al. 2013). Such findings highlight the potential of *Litchi chinensis* as a promising candidate for developing novel, plant‐based interventions for AD. To provide a clearer overview of the complex mechanisms involved in Alzheimer's disease and the potential multitargeted actions of *Litchi chinensis*, a schematic illustration summarizing the major pathological pathways and the proposed neuroprotective mechanisms of lychee‐derived compounds is presented in Figure 1. In the studies included in this review, *Litchi chinensis* was predominantly investigated as naturally derived extracts or isolated phytochemicals obtained from plant materials, particularly from seeds and fruit pulp, rather than synthetically synthesized compounds.

This review aims to consolidate current knowledge on the neuroprotective role of *Litchi chinensis* and its phytochemicals in AD. By summarizing evidence from in vitro, in vivo, and preclinical studies, the review highlights the mechanisms through which lychee seed and fruit extracts may attenuate AD pathology. Furthermore, it discusses the therapeutic potential and future perspectives of lychee‐derived compounds in the management of AD.

## 2 Methodology

### 2.1 Research Question Formulation

The present study was conducted as a structured narrative literature review to summarize the available preclinical evidence regarding the neuroprotective potential of *Litchi chinensis* in AD. To guide the literature search and selection process, a focused research question was formulated:

“What experimental evidence exists regarding the neuroprotective effects of *Litchi chinensis* and its phytochemicals in models of Alzheimer's disease, and through which molecular mechanisms do these compounds exert their protective effects?”

This question was developed based on the increasing interest in plant‐derived bioactive compounds as potential therapeutic agents for neurodegenerative disorders and the need to consolidate existing mechanistic evidence related to *Litchi chinensis*.

### 2.2 Search Strategy

A structured literature search was conducted in major scientific databases, including PubMed, Scopus, Web of Science, ScienceDirect, and Google Scholar, up to August 2025. The key search terms were selected based on the study objectives and an initial screening of relevant literature. Both Medical Subject Headings (MeSH) and free‐text terms were used to enhance the sensitivity of the search. The primary search term “Litchi chinensis” was combined with related keywords and their synonyms, such as “lychee,” “Alzheimer's disease” OR “AD” OR “dementia,” “cognitive function” OR “memory,” “neuroprotective” OR “neuroprotection,” “neurodegeneration,” “antiproliferation,” and “pharmacological activities.” Additionally, terms such as “in vivo” and “in vitro” were included to capture experimental studies. Boolean operators (AND, OR) were applied to appropriately combine search terms across databases. No restrictions were imposed on publication date or language. The retrieved studies were thoroughly screened and assessed based on their origin, dosage, experimental design, proposed mechanisms, key findings, and future recommendations.

Initially, a total of 764 records were screened from various databases. After evaluating titles, abstracts, and removing duplicates or irrelevant studies, 753 articles were excluded for reasons such as lack of relevance, missing data, or outdated information. Finally, only 11 studies met the inclusion criteria and were selected for analysis, focusing on their reported neuroprotective effects, as shown in Figure 1.

### 2.3 Inclusion Criteria

The evaluation incorporated the following selection criteria: (1) studies utilizing laboratory animals, tissue samples, or cell cultures; (2) investigations exploring the therapeutic properties of *Litchi chinensis*; (3) reported experimental, preclinical, or clinical evidence of *Litchi chinensis* or its bioactive compounds in relation to AD or neuroprotection; and (4) research examining neuroprotective effects of *Litchi chinensis* in AD

### 2.4 Exclusion Criteria

Studies were excluded if they (1) focused on unrelated pharmacological activities of Litchi chinensis (e.g., anti‐diabetic, anti‐cancer, antimicrobial) without relevance to neuroprotection; (2) were not available in full text; (3) were duplicate publications; and (4) were conference abstracts, editorials, or commentaries without substantial data.

## 3 Results and Discussion

### 3.1 Literature Review: Neuroprotective Effects of Litchi chinensis in Alzheimer's Disease

#### 3.1.1 Inhibition of Amyloid‐Beta (Aβ) Accumulation

One of the earliest and most studied events in AD is the accumulation of Aβ peptides (Sehar et al. 2022). These peptides are derived from the APP, a transmembrane protein that can undergo cleavage by two alternative enzymatic pathways (Haass and Selkoe 1993). However, in AD, APP is predominantly processed by β‐secretase (BACE1) and γ‐secretase, leading to the production of insoluble Aβ peptides, particularly Aβ40 and the more toxic Aβ42 variant (Tabaton and Tamagno 2007). These peptides aggregate into oligomers and fibrils, ultimately forming extracellular amyloid plaques (Stewart and Radford 2017). Oligomeric Aβ species are considered especially neurotoxic, as they disrupt synaptic signaling, impair neurotransmitter release, alter calcium homeostasis, and initiate downstream oxidative and inflammatory responses (de Felice et al. 2004). Several studies have reported that *Litchi chinensis* extracts and its bioactive compounds reduce Aβ levels and related markers. In triple‐transgenic AD mice, oligonol (0.25–0.50 mg/mL) (from litchi) treatment reduced APP, BACE1, and overall Aβ burden (Chen et al. 2021). Tang et al. (2018a) showed that seed extract (0.7–2.8 g/kg/day, i.g.) reduced Aβ accumulation compared with the vehicle‐treated group (*p* < 0.01) in male Sprague–Dawley rats, suggesting its potential to protect against Aβ‐related neurodegeneration and cognitive decline (Tang et al. 2018a). Xiong et al. (2020) reported that catechin, procyanidin A1, and procyanidin A2 significantly reduced amyloid beta (Aβ) levels in HT22 cells. The decrease in Aβ suggests potential neuroprotective effects relevant to Alzheimer's disease (Xiong et al. 2020). Choi et al. (2014) and Chen et al. (2021) reported that oligonol significantly reduced amyloid‐β (Aβ) levels in mice models of Alzheimer's disease (Chen et al. 2021; Choi et al. 2014). This inhibitory effect on Aβ accumulation may be associated with modulation of amyloidogenic processing pathways, including reduced expression of APP and β‐secretase (BACE1), which are key enzymes involved in Aβ production (Cole and Vassar 2008). By suppressing these pathways, *Litchi chinensis* constituents may contribute to limiting amyloid plaque formation and subsequent neuronal damage in Alzheimer's disease.

#### 3.1.2 Reduction of Tau Hyperphosphorylation and Neurofibrillary Tangle Formation

Parallel to amyloid pathology, abnormal hyperphosphorylation of tau protein is a defining feature of AD (Sajjad et al. 2018). Tau is a microtubule‐associated protein responsible for stabilizing axonal microtubules (Barbier et al. 2019). In AD, hyperactivation of kinases such as glycogen synthase kinase‐3β (GSK‐3β), cyclin‐dependent kinase‐5 (CDK5), and others leads to excessive tau phosphorylation (Liu et al. 2016). Hyperphosphorylated tau loses its ability to stabilize microtubules, causing cytoskeletal disintegration and the formation of insoluble paired helical filaments, which accumulate as intracellular neurofibrillary tangles (NFTs) (Pandey et al. 2025). This destabilization compromises axonal transport, impairs communication between neurons, and ultimately contributes to neuronal dysfunction and death (Kril et al. 2002). In Sprague–Dawley rats, dried seed extract (120–480 mg/kg/day, i.g.) downregulated GSK‐3β and tau protein, thereby reducing Alzheimer's‐related pathology (Sun et al. 2020). In this study, tau pathology was assessed using immunohistochemistry with anti‐tau antibodies followed by quantitative optical density analysis, while pathway‐related proteins were further analyzed by Western blot. The experimental design included a sham control group and an Aβ_25–35_‐induced AD model group, with donepezil used as a positive control comparator alongside three doses of *Litchi chinensis* seed fraction (Sun et al. 2020). Another study by Tang et al. (2018a) showed that litchi seed extract (0.7–2.8 g/kg/day, i.g.) reduced tau protein accumulation in Sprague–Dawley rats, providing neuroprotection against Alzheimer's‐related tauopathy (Tang et al. 2018a). Xiong et al. (2020) revealed that, in HepG2 and HT22 cells, phytochemicals such as catechin, procyanidin A1, and procyanidin A2 (1–100 µM) from litchi inhibited GSK‐3β. In this study, HepG2 cells were used to investigate signaling pathways associated with GSK‐3β regulation, while neuronal HT22 cells provided a model for neuronal responses. Tau‐related gene expression in this study was evaluated using RT‐PCR, which demonstrated reduced tau hyperphosphorylation following treatment with these compounds (Xiong et al. 2020). Moreover, in triple‐transgenic male mice, oligonol (0.25–0.50 mg/mL) reduced tau hyperphosphorylation, contributing to the mitigation of Alzheimer's‐related neuronal pathology and cognitive deficits (Chen et al. 2021).

#### 3.1.3 Suppression of Neuroinflammation and Cytokine Regulation

Neuroinflammation plays a central role in amplifying AD pathology (Heneka et al. 2015). Microglia, the resident immune cells of the brain, are activated in response to Aβ plaques and neuronal debris (Walker 2020). While initially protective, chronic microglial activation leads to sustained release of pro‐inflammatory mediators such as tumor necrosis factor‐alpha (TNF‐α), interleukin‐1β (IL‐1β), and interleukin‐6 (IL‐6) (Ogunmokun et al. 2021). Other cytokines, such as interferon‐gamma (IFN‐γ), C‐X‐C motif chemokine ligand 12 (CXCL12), and oncostatin M (OSM), promote neuroinflammation by activating glial cells and amplifying cytokine signaling, leading to neuronal damage and cognitive decline (West 2019).

These cytokines induce neuronal injury, disrupt synaptic plasticity, and further promote Aβ production, creating a vicious cycle of inflammation and neurodegeneration (Wang et al. 2015). This prolonged neuroinflammatory state is now recognized as both a consequence of amyloid and tau pathology and a driving force of disease progression (Garbuz et al. 2021). However, litchi and its extracted compound showed significant neuroprotective effects via modulation of inflammatory cytokines. In triple transgenic male mice, Oligonol (0.25–0.50 mg/mL) reduced pro‐inflammatory cytokines IFN‐γ and TNF‐α and increased anti‐inflammatory IL‐4. This modulation of neuroinflammation contributed to AD protection (Chen et al. 2021). In 5× FAD male mice, administration of Oligonol (50 mg/kg, p.o.) lowered levels of the inflammatory cytokines IFN‐γ, CXCL12, and OSM, reducing neuroinflammation and providing protection against AD (Jo et al. 2023). Another study by Tang et al. (2018) revealed that in BV‐2 cells, lychee seed fraction (LSF) polyphenols (0.469 µg/mL) decreased the release of pro‐inflammatory cytokines TNF‐α, IL‐1β, and IL‐6, helping to mitigate neuroinflammatory responses (Tang et al. 2018). LSF showed significant anti‐neuroinflammatory activity in BV‐2 cells at 0.12–0.48 mg/L. It reduced key inflammatory mediators including IL‐1β, TNF‐α, COX‐2, iNOS, and NF‐κB in Aβ_1–42_‐stimulated cells. These effects were statistically significant compared to the negative control (*p* < 0.05) (Zhao et al. 2018).

#### 3.1.4 Attenuation of Oxidative Stress and Enhancement of Antioxidant Defenses

Mitochondria, the primary energy‐generating organelles in neurons, are severely compromised in AD (Clemente‐Suárez et al. 2023). Aβ peptides accumulate within mitochondrial membranes, impairing respiratory chain complexes and leading to reduced ATP production (Leal et al. 2020). This dysfunction promotes excessive generation of reactive oxygen species (ROS), which cause oxidative damage to lipids, proteins, and nucleic acids (Ozougwu 2016). Lipid peroxidation alters membrane integrity, protein oxidation disrupts enzymatic function, and DNA damage compromises genomic stability (Dutta et al. 2018). Neurons, with their high metabolic demands, are particularly vulnerable to oxidative stress, and cumulative mitochondrial injury is believed to contribute significantly to synaptic loss and neuronal death (Tönnies and Trushina 2017). However, litchi extracts effectively protected against oxidative damage by enhancing the antioxidant defense system. In male mice, Oligonol (100 and 200 mg/kg, p.o.) decreased oxidative stress by reducing nitric oxide formation and lipid peroxidation. This enhancement of antioxidant defense contributed to AD protection (Choi et al. 2014). Jo et al. (2023) reported that in 5× FAD male mice, Oligonol (50 mg/kg, p.o.) boosted antioxidant defenses by upregulating *Gclc*, *Gpx1*, *Sod2*, *G6pdh*, and *Pepck*, helping to reduce oxidative stress and provide protection against AD (Jo et al. 2023).

#### 3.1.5 Prevention of Apoptosis and Neuronal Loss

Programmed cell death through apoptosis is another critical mechanism underlying neuronal loss in AD (Goel et al. 2022). Both Aβ and hyperphosphorylated tau have been shown to trigger apoptotic cascades by disrupting mitochondrial integrity and activating pro‐apoptotic signaling pathways (Sharma et al. 2021). Release of cytochrome c from damaged mitochondria activates caspase‐9, which in turn initiates downstream effector caspases such as caspase‐3, culminating in DNA fragmentation and cell death (Zhou et al. 2024). Additionally, prolonged neuroinflammation and oxidative stress amplify apoptotic signaling by upregulating pro‐apoptotic proteins like Bax and downregulating anti‐apoptotic proteins such as Bcl‐2 (Kumari et al. 2023). The cumulative activation of these pathways accelerates neuronal apoptosis, contributing significantly to cortical and hippocampal atrophy and the progressive cognitive decline observed in AD (Bazhanova and Kozlov 2024). However, litchi extract and its phytochemicals demonstrated notable protective effects against AD by inhibiting apoptosis and reducing neuronal loss. Wang et al. (2017) reported that dried seed extract exerted neuroprotective effects against Alzheimer's‐related apoptosis by increasing Bcl‐2 expression and improving the Bcl‐2/Bax ratio, while concurrently downregulating Bax. In vitro studies with PC12 cells demonstrated reduced caspase‐3 mRNA levels, indicating suppression of apoptotic pathways. Similarly, in vivo experiments in Sprague–Dawley rats showed reduced neuronal damage through comparable regulation of apoptotic markers (Wang et al. 2017). In the study by Tang et al. (2018), LSF polyphenols demonstrated anti‐apoptotic effects in BV‐2 microglial cells. Treatment significantly increased the expression of the anti‐apoptotic protein Bcl‐2, while reducing pro‐apoptotic markers such as Bax and the Bax/Bcl‐2 ratio. This shift toward cell survival indicated suppression of apoptosis. Additionally, decreased caspase‐related mRNA expression further confirmed inhibition of apoptotic signaling, suggesting that LSF polyphenols protect neuronal cells by modulating the balance between pro‐ and anti‐apoptotic pathways (Tang et al. 2018). LSF demonstrated anti‐apoptotic effects in BV‐2 cells (in vitro) at 0.12–0.48 mg/L. It increased Bcl‐2 expression while reducing Bax, caspase‐3, and cleaved PARP levels, thereby attenuating apoptosis in Aβ_1–42_‐induced cells compared to negative control (*p* < 0.05) (Zhao et al. 2018). LSP exhibited anti‐inflammatory effects in bEnd.3 cells (in vitro) at 40 µM and in APP/PS1 mice. It suppressed NLRP3 inflammasome activation along with downregulation of NLRP3, caspase‐1, and IL‐1β expression in Aβ_25–35_‐induced conditions. These findings indicate attenuation of neuroinflammation compared to saline‐treated controls (*p* < 0.05) (Xiong et al. 2021).

#### 3.1.6 Preservation of Synaptic Integrity and Cognitive Function

The convergence of amyloid accumulation, tau pathology, neuroinflammation, oxidative stress, and insulin resistance culminates in widespread synaptic dysfunction (Huang et al. 2025). Synapses are particularly vulnerable to toxic Aβ oligomers, which impair neurotransmitter release and long‐term potentiation (LTP), a cellular mechanism underlying memory formation (Tu et al. 2014). Progressive synaptic loss is strongly correlated with cognitive decline in AD patients. As disease advances, extensive neuronal death occurs, particularly in the hippocampus and cortex, leading to the profound memory loss, language deficits, and behavioral changes characteristic of clinical AD (Budson and Solomon 2021). However, litchi extract and compound significantly preserve synaptic integrity. Oligonol, derived from lychee (*Litchi chinensis*), enhanced synaptic integrity in AD mice by upregulating PSD95, synaptophysin, and serotonin receptor proteins, thereby supporting synaptic plasticity and neuronal communication. These effects help preserve cognitive function and reduce synaptic loss (Jo et al. 2023).

#### 3.1.7 Miscellaneous Protective Effects (Mitochondrial, ER Stress, and Neurotrophic Support)

In addition to the well‐studied mechanisms involving Aβ, tau, oxidative stress, and apoptosis, lychee‐derived compounds exhibited several other protective effects by modulating diverse molecular markers. Mitochondrial and proteasomal functions were enhanced through upregulation of NDUS1 and PSA6. NDUS1, a component of mitochondrial complex I, contributes to energy production, while PSA6 supports protein degradation via the ubiquitin proteasome system. These changes suggest that lychee compounds restore neuronal bioenergetics and protein homeostasis (Chen et al. 2021). ER stress, a significant contributor to neuronal death, was alleviated through regulation of GRP 78 and *Wfs1*. GRP78 serves as a key ER chaperone, and *Wfs1* is involved in ER stress resistance and calcium signaling. Their modulation indicates that lychee compounds reduce unfolded protein stress and support neuronal survival (Chen et al. 2021; Sakurai et al. 2013). Stress and signaling pathways were also modulated. Downregulation of JNK and ankyrin repeat domain 11 (*Ankrd11*), along with regulation of guanine nucleotide‐binding protein 1, indicated that lychee compounds attenuate stress‐activated signaling and restore G‐protein–mediated pathways, both of which are disrupted in AD (Sakurai et al. 2013). Neurotrophic and metabolic support were evidenced by upregulation of IGF‐2 and FGF21, growth factors critical for neuronal survival, glucose metabolism, and synaptic integrity. Similarly, TIMP‐1, an extracellular matrix regulator, was increased, suggesting protection against excessive metalloproteinase activity and preservation of the neuronal environment (Jo et al. 2023).

**FIGURE 2:**
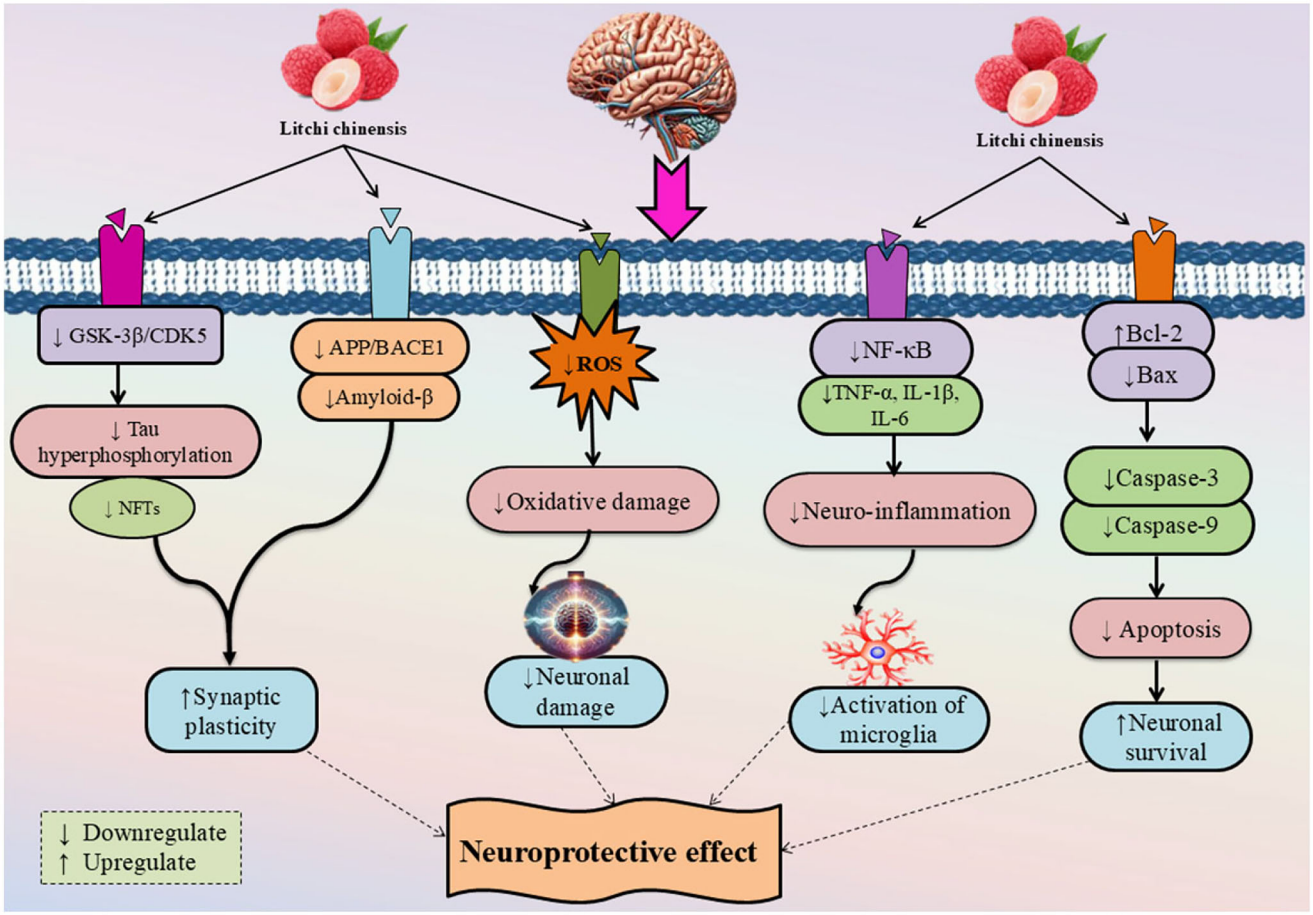
Neuroprotective mechanism of *Litchi chinensis* against Alzheimer's disease. The mechanism was schematically illustrated using Microsoft PowerPoint (2013 version), with graphical assets and figures sourced from the following publicly available repositories: https://smart.servier.com/, https://www.biorender.com/, https://www.pngwing.com/, and https://www.pngegg.com/. The figure illustrates the neuroprotective effects of *Litchi chinensis* on neuronal health through a pathway diagram, showing its influence on cellular processes. *Litchi chinensis* downregulates GSK‐3β/CDK5, reducing tau hyperphosphorylation and enhancing synaptic plasticity, while also decreasing APP/BACE1 activity to lower amyloid‐β production. It upregulates Bcl‐2 and downregulates Bax, caspase‐3, and caspase‐9, promoting neuronal survival and inhibiting apoptosis. ROS contributes to oxidative and neuronal damage, but *Litchi chinensis* mitigates this by downregulating NF‐κB, which reduces the production of pro‐inflammatory cytokines TNF‐α, IL‐1β, and IL‐6, thereby decreasing neuroinflammation and microglia activation. The converging pathways highlight a neuroprotective effect, supporting neuronal health and function.

Overall study demonstrated that *Litchi chinensis* extracts and their bioactive compounds exhibit multifaceted neuroprotective effects against AD. They inhibit Aβ accumulation by downregulating APP and BACE1 and reduce tau hyperphosphorylation via GSK‐3β inhibition, thus preventing plaque and tangle formation. Anti‐inflammatory effects are achieved through suppression of cytokines like TNF‐α, IL‐1β, and IFN‐γ, while enhancing anti‐inflammatory IL‐4. Antioxidant defense is boosted by upregulating enzymes such as *Gpx1*, *Sod2*, and *G6pdh*, reducing oxidative damage. Apoptosis is suppressed through increased Bcl‐2 and reduced Bax and caspase activity, protecting neurons from programmed cell death. Synaptic integrity is preserved by enhancing PSD95 and synaptophysin, supporting cognitive function. Additional benefits include improved mitochondrial and proteasomal function (NDUS1, PSA6), alleviation of ER stress (GRP78, *Wfs1*), and upregulation of neurotrophic factors IGF‐2 and FGF21. Together, these effects demonstrate that lychee‐derived compounds protect against AD by inhibiting its underlying pathophysiological processes. All the summarized data are presented in Table 1, while the protective mechanisms against Alzheimer's disease are illustrated in Figure 2.

**TABLE 1:** Neuroprotective mechanism of *Litchi chinensis* against Alzheimer's disease.

Extract type/ phytochemical's name	Cell line/mice model	Dose/ concentration (R/A)	Results/mechanism	AD induction	Controls	Significance	References
Dried extract (seed)	Sprague–Dawley (SD) rats (*n* = 10), in vivo	120‒480 mg/kg/d (i.g.)	↑AKT, cognitive functions ↓GSK‐3β, tau protein	Aβ_25–35_	NS (negative control), donepezil (positive control)	*p* < 0.01, *p* < 0.05, *p* < 0.001	Sun et al. 2020
Seed extract	Male Sprague– Dawley rats, in vivo	0.7− 2.8 g/kg/day (i.g.)	↑ Cognitive functions, ↓Neuronal injury, Aβ, AGEs, and Tau protein	T2DM‐induced cognitive impairment (similar to pathological mechanism)	NS (negative control), donepezil (positive control)	*p* < 0.01	Tang et al. 2018a
Catechin, procyanidin A1 and procyanidin A2	HepG2, HT22 cell lines, in vitro	1‒100 µM	↑ IRS‐1/PI3K/Akt ↓GSK‐3β, hyperphosphorylated Tau, Aβ	Aβ_25–35_	NS (negative control), donepezil (positive control)	*p* < 0.05	Xiong et al. 2020
Dried extract (seed) LSS	PC12 cell line in vitro	0.95−7.60 mg/L	↑Cognitive function, protein expression of Bcl‐2, Bcl‐2/Bax ↓Apoptosis, Bax, caspase‐3, neuronal injury	Aβ_25–35_	NS (negative control), donepezil (positive control)	*p* < 0.01	Wang et al. 2017
	male Sprague–Dawley (SD), in vivo	120−480 mg/kg (i.g.)					
Oligonol	Male mice (*n* = 5), in vivo	100 and 200 mg/kg (p.o.)	↑ Recognition ability, spatial cognition ability, spatial learning, memory function ↓ Nitric oxide formation, lipid peroxidation, Aβ	Aβ_25–35_	NaCl + water	*p* < 0.05 or *p* < 0.01	Choi et al. 2014
Oligonol	5 × FAD male mice (*n* = 3), in vivo	50 mg/kg (p.o.)	↑Cognitive function, antioxidant response, mRNA levels, *Gclc, Gpx1, Sod2, G6pdh*, *Pepck*, *Pgc1α*, Blc, TIMP‐1, IGF‐2, FGF21, *PSD95*, *SYP*, GFAP, c‐Fos, protein synaptic plasticity, neuronal function, glial activation ↓ Anxiety, IFN‐γ, CXCL12, OSM, ROS	Genetic modification (↑Aβ plaque, cognitive impairment, anxiety‐like behavior)	Water (negative control)	*p* < 0.05	Jo et al. 2023
Oligonol	Triple transgenic male mouse, (*n* = 12), in vivo	0.25–0.50 mg/mL	↑IL‐4, NDUS1, synapsin II level, PSA6 level ↓Cognitive deficits, APP, BACE1, Aβ level, tau hyper‐phosphorylation, IFN‐γ, TNFα, GRP 78, mean vimentin expression, DC1I1	3xTg‐AD	—	*p* < 0.05	Chen et al. 2021
Oligonol	NG108—15 cells, in vitro	2 mg/mL	↑*Wfs1*, *Cbln4*, lamin B1, matrix Gla protein, JNK ↓Cognitive impairment, ankyrin repeat domain 11, guanine nucleotide‐binding protein, polypeptide 1, ER stress	Natural accelerated senescence model (↑Aβ overproduction, tau hyperphosphorylation, cognitive decline)	SAMR1 mice (positive control) untreated SAMP8 mice (negative control)	*p* < 0.05	Sakurai et al. 2013
	Male SAMP8 mice (*n* = 6), in vivo	100 mg/kg (orally)					
LSF (polyphenols)	BV‐2 cell line, in vitro	0.469 µg/mL	↑ Bcl‐2 ↓Apoptosis, Bax/Bcl‐2 ratio, mRNA levels, release of (TNF‐α, IL‐1β, IL‐6,) NF‐κB, Bax	Aβ_1–42_	BV‐2 cells (negative control) Aβ‐treated cells (positive control)	*p* < 0.05	Tang et al. 2018
Lychee seed fraction (LSF)	BV‐2 cell line, in vitro	0.12–0.48 mg/L	↑Bcl‐2 expression ↓IL‐1β, TNF‐α, COX‐2, iNOS, NF‐κB, Bax, caspase‐3, cleaved‐PARP expression, apoptosis, inflammatory cytokine	Aβ_1–42_	BV‐2 cells (negative control)	*p* < 0.05	Zhao et al. 2018
Lychee seed polyphenol (LSP)	bEnd.3 cell line, in vitro	40 µM	↑Autophagy, AMPK expression, ULK1 expression, spatial learning and memory function ↓BBB, NLRP3 inflammasome activation, NLRP3 expression, caspase‐1 expression, IL‐1β expression, p62 expression, mTOR expression	Aβ_25–35_	Saline (negative control)	*p* < 0.05	Xiong et al. 2021
	APP/PS1 mice, in vivo	0–64 µg/mL					

↑: increase; ↓: decrease; AKT: protein kinase B; Blc: B lymphocyte chemoattractant; DXM: dexamethasone; FGF21: fibroblast growth factor 21; Gclc: glutamate‐cysteine ligase catalytic; G6pdh: glucose 6‐phosphate dehydrogenase; Gpx1: glutathione peroxidase 1; i.g.: intragastric; IFN‐γ: Interferon gamm; CXCL12:  chemokine ligand C–X–C motif chemokine ligand 12; IGF‐2: insulin‐like growth factor 2; IL‐1β: interleukin‐1β; IL‐6: interleukin‐6; LSF: lychee seed fraction; LSS: lychee seed saponins; MDA: measurement of malondialdehyde; NF‐κB: nuclear factor‐κB; OSM: oncostatin M; p.o.: per orally; Pepck: phosphoenolpyruvate carboxykinase; Pgc1α: peroxisome proliferator‐activated receptor‐c coactivator‐1a, ROS: reactive oxygen species; SD: Sprague–Dawley; Sod2: superoxide dismutase 2; TIMP‐1: tissue inhibitor of metalloproteinase‐1; TNF‐α: tumor necrosis factor‐α.

### 3.2 Limitations and Challenges

Despite the promising neuroprotective potential of *Litchi chinensis*, several limitations should be considered. Most of the available evidence is derived from in vitro studies and animal models, while clinical investigations in humans remain limited. In addition, variations in extraction methods, plant parts used, and phytochemical composition may lead to inconsistencies in reported biological activities. The optimal dosage, pharmacokinetic profile, and long‐term safety of *Litchi chinensis*–derived compounds have not been fully established. Furthermore, some studies have used different experimental models and analytical methods, which may affect the comparability of results across studies. These limitations highlight the need for more standardized and clinically relevant research to fully validate the therapeutic potential of *Litchi chinensis* in Alzheimer's disease. Another limitation of this review is that the toxicity and safety profile of *Litchi chinensis*–derived compounds was not systematically evaluated. Although some individual studies reported acceptable tolerability in experimental models, a comprehensive assessment of toxicity, dose‐dependent safety, and long‐term effects was beyond the scope of the present review. Another limitation of this review is the lack of quantitative data or percentages in the included studies, as most of the available research primarily reported qualitative outcomes. This review did not strictly apply a structured PICOS (Population, Intervention, Comparison, Outcomes, Study design) framework during study selection, as it was conducted as a narrative synthesis. As a result, some heterogeneity in study characteristics and outcome reporting may exist. In addition, a formal risk of bias assessment was not performed, which may limit the ability to fully evaluate the internal validity and methodological quality of the included studies

### 3.3 Future Research Perspectives

Future studies should focus on conducting well‐designed clinical trials to confirm the neuroprotective effects of *Litchi chinensis* in humans. Further investigation is also required to identify and characterize the specific bioactive compounds responsible for its therapeutic effects and to elucidate their precise molecular mechanisms. Again, more studies should focus on providing standardized quantitative data to allow clearer comparison and stronger evaluation of the neuroprotective effects. Standardization of extraction procedures, dosage regimens, and experimental models will improve reproducibility and facilitate comparisons between studies. In addition, research on pharmacokinetics, bioavailability, and long‐term safety and toxicity profiles is essential to determine the clinical applicability of *Litchi chinensis*–derived compounds. Exploring synergistic interactions between phytochemicals and potential combination therapies with existing anti‐Alzheimer's drugs may also provide new avenues for developing effective therapeutic strategies. Future research should adopt a well‐defined PICOS framework to ensure more systematic and transparent study selection. Additionally, the use of standardized risk of bias assessment tools (e.g., SYRCLE, Cochrane RoB) is strongly recommended to improve the methodological rigor, reproducibility, and reliability of evidence synthesis in this area.

## 4 Conclusion

*Litchi chinensis* and its phytochemicals exhibit remarkable neuroprotective potential against AD by acting through multiple pathways. It reduces Aβ accumulation and tau hyperphosphorylation, thereby preventing plaque and tangle formation. Its extracts suppress neuroinflammation by regulating cytokines and enhance antioxidant defenses to counter oxidative damage. Additionally, litchi compounds inhibit apoptosis by balancing pro‐ and anti‐apoptotic proteins, while also preserving synaptic integrity and improving cognitive functions in preclinical studies. Beyond these effects, they support mitochondrial activity and promote neurotrophic factors, contributing to overall neuronal health. Collectively, these findings highlight Litchi chinensis as a promising natural therapeutic option for the prevention and management of AD. However, most findings are limited to preclinical models, and translational studies remain scarce. Standardized extracts, bioavailability improvement, and nanotechnology‐based delivery systems may enhance clinical outcomes. Future research must focus on pharmacokinetics, long‐term safety, and well‐designed clinical trials. Overall, *Litchi chinensis* represents a promising natural candidate for developing novel interventions against AD.

## Author Contributions

**Asif Khan Sherwani**: writing – review and editing, visualization, software, validation. **Mohammad Y. Alshahrani**: conceptualization, writing – original draft, writing – review and editing, formal analysis, supervision. **Uzma Asif**: writing – review and editing, validation, software. **Samy Selim**: investigation, validation, visualization, writing – review and editing. **Md. Arif Hossain**: conceptualization, writing – original draft, writing – review and editing. **Asmaul Husna Bristy**: investigation, software, resources, visualization. **Rakib Hossan**: conceptualization, investigation, methodology, validation, software. **Md. Sakib Al Hasan**: conceptualization, writing – original draft, writing – review and editing, visualization, formal analysis, project administration, supervision, resources. **Emon Mia**: conceptualization, writing – original draft, writing – review and editing. **Imam Hossen Rakib**: conceptualization, writing – original draft, writing – review and editing. **Noshin Tasnim Yana**: investigation, validation, methodology, software, resources, data curation, visualization. **Khadija Akter**: conceptualization, writing – original draft, writing – review and editing, formal analysis, project administration, supervision. All authors have read and agreed to the published version of the manuscript.

## Funding

The authors have nothing to report.

## Ethics Statement

This article is a review of published literature and does not involve any new studies with human participants or animals performed by any of the authors. Therefore, no specific ethical approval was required.

## Conflicts of Interest

The authors declare no competing interests.

## Data Availability

The data that support the findings of this study are available from the corresponding author upon reasonable request.
